# Experimental study on instability and failure mechanism of sandstone under freeze-thaw and load

**DOI:** 10.1371/journal.pone.0323484

**Published:** 2025-05-23

**Authors:** Wenyu Lv, Ru You, Chuangye Wang, Zhihe Wang, Yongping Wu, Panshi Xie, Chao Lyu, Sheng Luo, Yichen Li

**Affiliations:** 1 College of Energy and Mining Engineering, Xi’an University of Science and Technology, Xi’an, China; 2 College of Mining and Coal, Inner Mongolia University of Science and Technology, Baotou, China; 3 School of Chemical Engineering, University of Adelaide, Adelaide, Australia; 4 Sichuan Coal Group Huarong Energy Co., Ltd. Taiping Coal Mine, Panzhihua, China; King Mongkut's University of Technology North Bangkok, THAILAND

## Abstract

Freeze-thaw damage is the primary cause of instability in rock slopes in cold regions, while the mechanical properties of rock are significantly influenced by loading rate. This study aims to investigate the evolution of mechanical behavior of sandstone under the coupled effects of loading rate and freeze-thaw cycles. Uniaxial compression tests were conducted on sandstone specimens subjected to different numbers of freeze-thaw cycles (0, 30, 50, 70) and loading rates (0.05, 0.10, 0.15, 0.20 mm/min) to systematically study the deterioration mechanism of its mechanical properties. The experimental setup incorporated a real-time acoustic emission (AE) monitoring system combined with high-speed camera technology to analyze the influence of loading rate and freeze-thaw cycles on the mechanical characteristics, failure modes, and AE features of sandstone. The results demonstrate that the uniaxial compressive strength (UCS) and elastic modulus (*E*) of sandstone exhibit a negative correlation with the number of freeze-thaw cycles and a positive correlation with loading rate, while the ductility characteristics show an opposite trend. Notably, the attenuation constant λ follows a monotonically decreasing pattern with increasing loading rate. Failure mode analysis reveals that sandstone predominantly exhibits tensile-shear composite failure characteristics at complete failure, with tensile cracks dominating in quantity. As the loading rate increases, the proportion of tensile cracks significantly rises, whereas the increase in freeze-thaw cycles reduces the disparity between the number of shear and tensile cracks. The initial stage of internal crack propagation in sandstone is accompanied by a significant increase in AE events rate and drastic fluctuations in *b*-value. The absence of low AE events rate and the sharp decline in *b*-value can serve as crucial precursor indicators for predicting the instability and failure of sandstone. Based on the experimental data, a predictive model for the strength index attenuation of freeze-thaw damaged sandstone considering loading rate effects was established, providing a theoretical basis for stability assessment of rock engineering in cold regions.

## 1. Introduction

Inner Mongolia is located between 37°24′ and 53°23′ N, characterized by significant seasonal and diurnal temperature variations, as well as abundant mineral resources. During mining operations in permafrost regions, water within the discontinuities of rock undergoes volume expansion due to phase transitions, generating frost heave pressure [1]. This pressure further promotes the propagation of new cracks, leading to the deterioration of rock mechanical properties, reduced slope stability, and an increased risk of geological hazards such as landslides.

In recent years, extensive research has been conducted on the physical properties of rocks subjected to freezing and thawing. An increase in freeze-thaw cycles reduces the strength and elastic modulus (*E*) of the rock [2,3], and leads to a decline in the internal friction angle and cohesion [4]. This process accelerates the shedding of surface particles [5], promotes the expansion of rock pores [6], increases effective porosity, and results in the formation of new cracks dominated by larger pores [7,8]. Additionally, it reduces the fractal dimensionality and complexity of the pore structure [9], along with a decrease in P-wave and ultrasonic pulse velocities [10,11]. The extent of rock deterioration due to freeze-thaw cycles correlates with the number of cycles, but the effect is less pronounced in rocks with smaller initial pore sizes and higher uniaxial compressive strength (UCS) [12,13]. Moreover, a greater temperature fluctuation range intensifies the degradation of rock mechanical properties [14,15]. Water saturation also significantly affects freeze-thaw damage, with higher water content leading to more pronounced frost heave effects and greater damage [16]. Furthermore, the impact of freeze-thaw cycles varies across different chemical solutions, with rock deterioration in acidic solutions being more severe than in alkaline environments [17].

The mechanical properties of slope rock in cold mining regions are influenced not only by climatic conditions but also by the loading rate experienced during mining operations. As a potential risk factor for inducing rock mass instability, this issue has garnered significant attention and in-depth investigation from researchers worldwide in recent years [18,19]. Numerous studies have demonstrated that an increase in loading rate enhances rock strength and *E* [20], intensifies crack propagation, increases the number of rock fragments generated during failure, and reduces fragment size [21]. Under high loading rates, the growth rate of dissipated energy decreases, resulting in lower energy utilization efficiency and an increased energy requirement for rock fragmentation [22,23]. He et al. [24] investigated the damage evolution of sandstone under different loading rates and found that at lower loading rates, microcracks develop more extensively, leading to more severe damage. Similarly, Lajtai et al. [25] compared the strength of brittle limestone and ductile salt rock, revealing that loading rate effects are more pronounced in ductile salt rock than in brittle limestone. However, rock strength does not continuously increase with loading rate; instead, a critical loading rate exists beyond which further increases no longer significantly enhance rock strength and *E* [26]. Current research on crack mechanisms under different loading rates primarily focuses on mechanical properties and energy evolution, while studies employing alternative analytical approaches remain relatively limited. Additionally, investigations on the effects of loading rate on rock mechanical behavior often overlook its coupling with other influencing factors, which warrants further exploration.

Acoustic emission (AE) technology allows for the real-time recording of elastic wave signals generated during the damage and failure processes of rocks. Changes in AE parameters and their waveform characteristics are closely related to the internal damage evolution of rocks [27–29]-[30], which is why this technology has been widely applied to study the damage evolution in rocks under stress [31,32]. Zhao et al. [33] established the relationship between axial stress, strain, AE event rate, and average centroid frequency, demonstrating that AE signals are closely associated with the changes in axial stress and strain in sandstone specimens. Derivative parameters, which can be directly or indirectly calculated from AE parameters, effectively characterize the rupture patterns of rocks. RA and AF are the most common indicators for distinguishing different types of cracks [34]. Based on phase space reconstruction theory, Li et al. [35] analyzed the fractal characteristics of AE signals at various stages of rock failure using the Grassberger-Procaccia algorithm. Aker et al. [36] studied the shear and tensile failure-related AE around horizontal boreholes under triaxial stress conditions, combining AE event rate with macroscopic deformation observations, and predicted possible failure mechanisms through the analysis of the isotropic and deviatoric components of seismic moment tensors.

Numerous studies have explored the deterioration mechanisms of rock mechanical properties under freeze-thaw cycles or at loading rates. However, a systematic investigation into the influence of varying loading rates under different freeze-thaw cycles on rock mechanical stability remains limited. Based on previous experimental studies [37,38]-[39], this paper conducts uniaxial compression tests on sandstone subjected to different numbers of freeze-thaw cycles at varying loading rates. Non-destructive monitoring was performed using AE and high-speed imaging to analyze the mechanical properties, damage patterns, and damage evolution of the rock. Furthermore, a strength attenuation model for freeze-thaw sandstone under different loading rates was established to elucidate the instability and failure mechanisms under the coupled effects of these two factors. The findings of this study provide theoretical support and computational references for the stability assessment, disaster prediction, and risk evaluation of engineering rock masses in cold regions, while also contributing to the protection of human safety and property security.

## 2. Materials and method

### 2.1 Sample preparation

Sandstone, a common rock type found in mining operations in Inner Mongolia, China, is characterized by its high water absorption and significant gravel content. For this experiment, the sandstone was collected from an open pit mine in Inner Mongolia. Using the same block for sample preparation minimized the variability in internal composition. First, core samples were drilled using a ZS-200 core drilling machine, then cut flat at both ends with a cutting machine, and finally polished with an SHM-200 grinder to produce standard cylindrical specimens with a diameter of 50 mm and a height of 100 mm. The accuracy of the sample meets the standards recommended by the International Society of Rock Mechanics and Engineering (ISRM) test regulations, ensuring that height and diameter deviations did not exceed 0.3 mm and that the flatness deviation of both ends remained within 0.25°.

### 2.2 Experimental procedure

To simulate the actual climatic conditions of open-pit engineering in Inner Mongolia, water-saturated sandstone samples were subjected to freeze-thaw cycles. Each cycle consisted of freezing at -30°C for 4 hours, followed by immersion in water at 30°C for another 4 hours. The sandstone samples underwent 0, 30, 50, and 70 freeze-thaw cycles, after which uniaxial compression tests were conducted at different loading rates (0.05, 0.10, 0.15, and 0.20 mm/min), resulting in a total of 16 experimental groups. Each group was tested three times to ensure data reliability.

**Fig 1:**
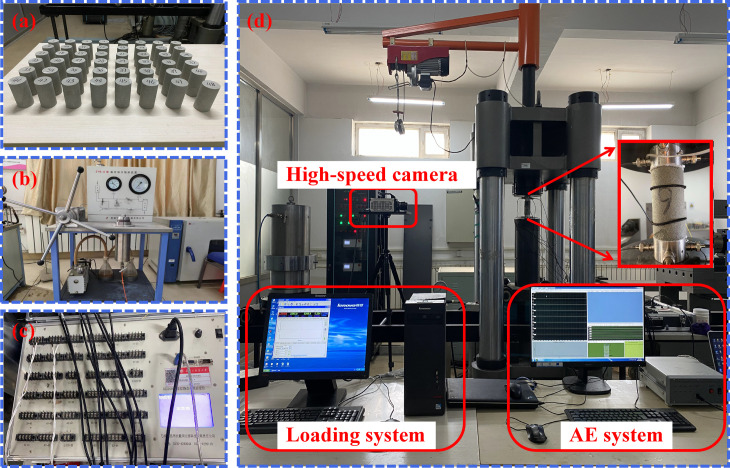
(a) 48 sandstone samples used in this experiment, (b) ZYB-II vacuum pressurized water saturation device, (c)static strain gauge, (d) diagram of experimental system, including uniaxial compression testing system, AE system and high-speed camera.

To minimize errors caused by rock heterogeneity, sandstone samples were grouped based on their porosity, T₂ values, and wave velocity. A total of 48 sandstone samples (Fig 1(a)) were fully saturated using the ZYB-II vacuum pressurized water saturation device (Fig 1(b)) before undergoing freeze-thaw cycling. After completing the freeze-thaw cycles, the sandstone samples were connected to a BZ-BZ2205C static strain gauge (Fig 1(c)) and a DS-5 acoustic emission system (Fig 1(d)). Coupling agents were applied between the acoustic emission sensors and the sandstone surface to minimize noise interference. The prepared samples were then placed on the loading platform, preloaded to 2 kN using the SAS-2000 uniaxial compression testing system (Fig 1(d)). while an HX-7s high-speed camera was employed to capture the evolution of external cracks.

During the test, the uniaxial compression system applied axial compression to the sandstone at the designated loading rates until complete failure. The uniaxial compression system, AE system, and high-speed camera were activated and deactivated simultaneously to record the stress-strain characteristics of the sandstone, AE signals, and the evolution of external cracks throughout the compression process. The test setup is shown in Fig 1.

## 3. Mechanical properties and failure process results

### 3.1 Analysis of mechanical properties characters

**Fig 2:**
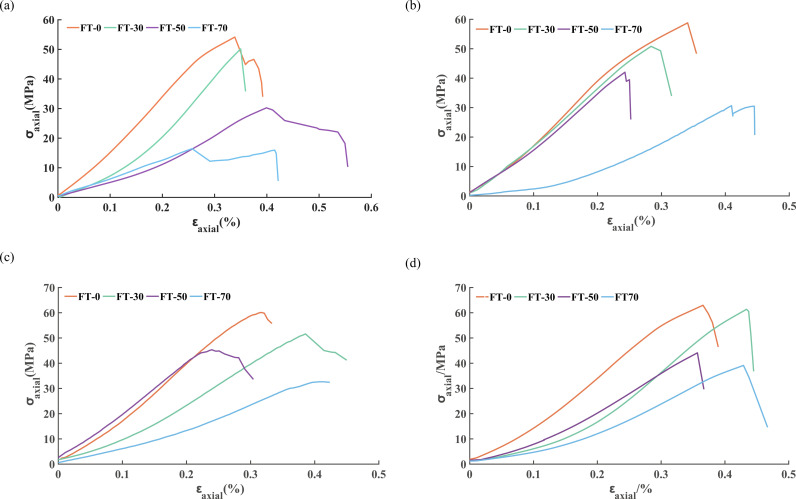
Stress-strain curves of freeze-thaw cycling sandstone under different loading rates. The sandstone samples subjected to 0, 30, 50, and 70 freeze-thaw cycles were labeled as FT0, FT30, FT50, and FT70, respectively. (a) 0.05 mm/min, (b) 0.10 mm/min, (c) 0.15 mm/min, (d) 0.20 mm/min.

Fig 2 displays the stress-strain curves of freeze-thaw sandstone samples subjected to various loading rates. In general, freeze-thaw cycles have the most significant effect on the UCS of the sandstone. The UCS decreases with the increase of freeze-thaw cycles, and the deformation of sandstone increases with the increase of freeze-thaw cycles when reaching complete failure. Under the same number of freeze-thaw cycles, an increase in the loading rate results in higher stress levels and bearing capacity in the sandstone, whereas the deformation observed in the post-peak residual stage decreases.

**Fig 3:**
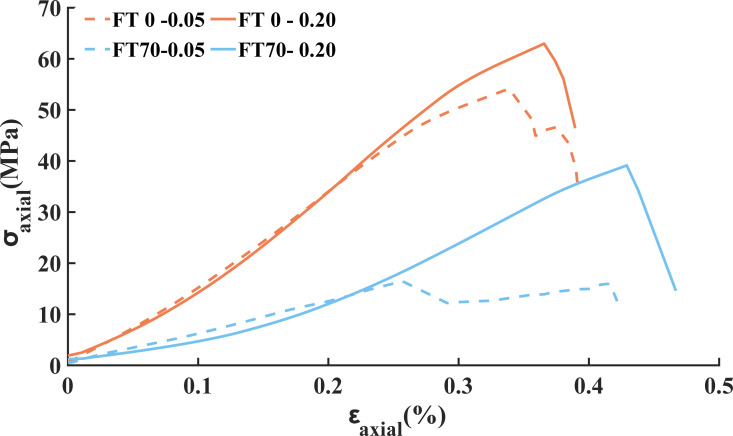
Stress-strain curves of four groups of typical sandstone samples.

The stress-strain curves of four groups of typical sandstone samples, subjected to loading rates of 0.05 mm/min and 0.20 mm/min and freeze-thaw cycles of 0 and 70, are shown in Fig 3. It can be observed that the deformation of unfrozen sandstone shows little difference at complete failure under different loading rates. The UCS of sandstone is the highest for 0 freeze-thaw cycles under a loading rate of 0.20 mm/min, and the lowest for 70 freeze-thaw cycles with a loading rate of 0.05 mm/min. An increase in the loading rate somewhat mitigates the impact of freeze-thaw cycles on the UCS.

The mechanical parameters of sandstone samples subjected to different freeze-thaw cycles at loading rates of 0.05 mm/min and 0.20 mm/min are presented in Table 1. When the loading rate is 0.05 mm/min, the UCS decreases by 69.57% after 70 freeze-thaw cycles, whereas at a loading rate of 0.20 mm/min, the decrease is 37.86%. The *E* of sandstone decreases with an increase in freeze-thaw cycles and increases with a higher loading rate. Table 1 illustrates the decline in the *E* of sandstone across different freeze-thaw cycles at both loading rates, highlighting the significant impact of freeze-thaw cycles on the *E*. This impact intensifies as the number of freeze-thaw cycles increases. While the loading rate has a minimal effect on the *E* of unfrozen sandstone, its influence on the *E* becomes more pronounced with an increase in freeze-thaw cycles.

**Table 1:** Detailed table of sandstones mechanical parameters change.

Group Number	0.05 mm/min				0.2 mm/min			
	UCS(MPa)	Decrease (%)	*E* (GPa)	Decrease (%)	UCS(MPa)	Decrease (%)	*E* (GPa)	Decrease (%)
FT0	54.15	—	19.60	—	62.97	—	21.24	—
FT30	50.16	7.37	15.46	21.12	61.38	2.52	21.21	0.14
FT50	34.62	36.07	12.54	36.02	44.11	29.95	15.46	27.21
FT70	16.48	69.57	6.78	65.41	39.13	37.86	12.06	43.22

### 3.2 Exponential decay model analysis

Mut-lutürk et al. [40] initially introduced an exponential decay model, while Jamshidi et al. [41] evaluated the durability of rock materials by calculating decay constants and half-lives, as illustrated in Eq. (1). This model is useful for elucidating the reduction in sandstone strength attributable to freeze-thaw cycles [2].


$$I_{n}=I_{0}e^{-\lambda n}$$ (1)

where *n* is the number of freeze-thaw cycles; *I*_*n*_ and *I*_*0*_ are the UCS of rock after *n* and 0 freeze-thaw cycles, respectively; and λ is the attenuation constant, indicating the characteristic parameter of frost resistance of rock. A larger λ, indicates a more serious deterioration of sandstone.

**Fig 4:**
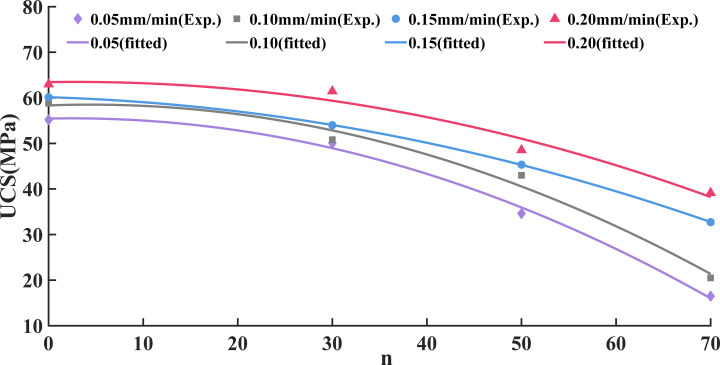
Variation law of UCS.

The changes in the UCS of sandstone under different loading rates and freeze-thaw cycles are shown in Fig 4.

By analyzing and fitting the test data, the strength index attenuation model of freeze-thaw sandstone under different loading rates is obtained, and the fitted equation is shown in Eqs. (2)-(5):


$$I_{n(0.05)}=54.19\times e^{\left(-0.009960n\right)},R^{2}=0.9049$$ (2)

$$I_{n(0.10)}=58.80\times e^{\left(-0.009012n\right)},R^{2}=0.8565$$ (3)

$$I_{n(0.15)}=60.14\times e^{\left(-0.006759n\right)},R^{2}=0.9213$$ (4)

$$I_{n(0.20)}=62.97\times e^{\left(-0.005308n\right)},R^{2}=0.8763$$ (5)

Where, *I*_*n*_(0.05)、*I*_*n*_(0.10)、*I*_*n*_(0.15) and *I*_*n*_(0.20) are respectively the UCS of sandstone after n freeze-thaw cycles at loading rates of 0.05, 0.10, 0.15 and 0.20 mm/min, respectively, MPa. The constants 54.19, 58.80, 60.14 and 62.97 are the UCS of sandstone with 0 freeze-thaw cycles at loading rates of 0.05, 0.10, 0.15 and 0.20 mm/min, respectively, MPa.

It can be seen from Eqs. (2)-(5) that the attenuation constant λ is 0.00996, 0.009012, 0.006759, and 0.005308 at loading rates of 0.10, 0.15, 0.20, and 0.25 mm/min, respectively. Additionally, the attenuation constant λ decreases as the loading rate increases.

### 3.3 Failure modes analysis of sandstone

**Fig 5:**
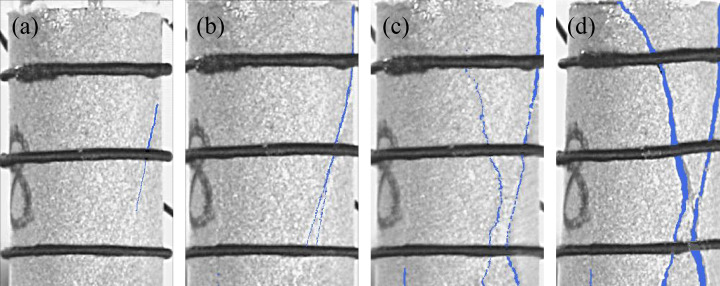
Crack characteristics of sandstone subjected to 0 freeze-thaw cycles at loading rate of 0.05mm/min. (a) *t*_*p*_-49.19s, (b) *t*_*p*_-12.03s, (c) *t*_*p*_, (d) *t*_*p*_ + 19.13s.

Four groups of typical sandstone crack characteristic images are shown in Fig 5-Fig 8. Select the images that exhibit crack development characteristics when sandstone cracks appear, develop, extend, and when the structure is completely penetrated. Analyze the crack characteristics of sandstone based on these images. In this Section, the time corresponding to the peak stress is taken as the reference point and define as $t_{p}$. The time associated with images exhibiting crack development characteristics will be represented relative to $t_{p}$.

**Fig 6:**
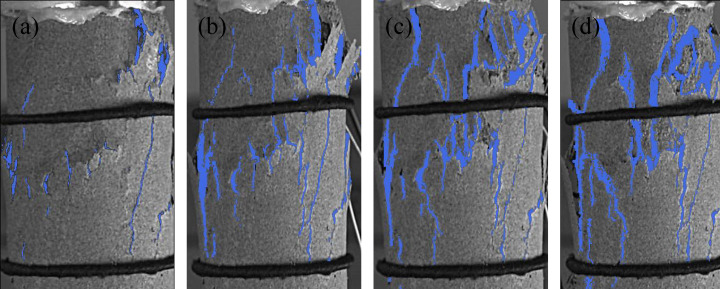
Crack characteristics of sandstone subjected to 70 freeze-thaw cycles at a loading rate of 0.05 mm/min. (a) *t*_*p*_-100.01s, (b) *t*_*p*_-19.89s, (c) *t*_*p*_, (d) *t*_*p*_ + 33.15s.

**Fig 7:**
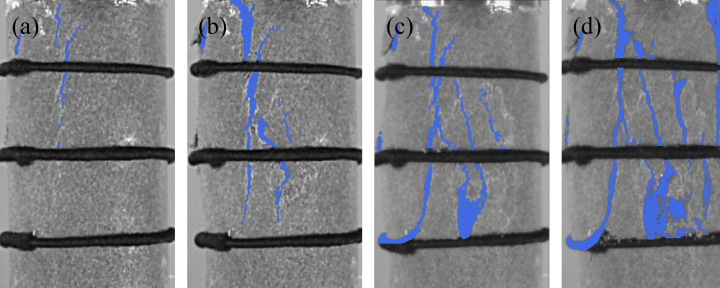
Crack characteristics of sandstone subjected to 0 freeze-thaw cycles at loading rate of 0.20 mm/min. (a)*t*_*p*_-29.84s, (b) *t*_*p*_-5.32s, (c) *t*_*p*_-3.55, (d) *t*_*p*_.

As shown in Fig 5, an obvious shear crack emerges in the sandstone with 0 freeze-thaw cycles at a loading rate of 0.05 mm/min, occurring 49.19 seconds prior to the peak stress. The crack then evolves into an X-shaped formation 12.03 seconds before the peak stress. At peak stress, the crack widens and is filled with sandstone debris. 19.13 seconds after the peak, the sandstone becomes unstable and disintegrates along the crack.

During the freeze-thaw cycles, the decrease in temperature creates a significant temperature gradient between the surface and the interior of the sandstone, leading to differential contraction due to the varying rates of thermal expansion and contraction between the surface and the interior. This results in the generation of tensile stress on the sandstone surface. Simultaneously, the freezing of water within the pores produces expansive forces, further exacerbating the stress state of the rock. The combined action of tensile stress and freezing expansion forces, after 70 freeze-thaw cycles, ultimately leads to particle detachment on the sandstone surface.

Under a loading rate of 0.05 mm/min, the sandstone subjected to 70 freeze-thaw cycles is shown in Fig 6. As stress increases, cracks on the sandstone surface extend from the initial freeze-thaw cracks. 100.01 seconds before reaching peak stress, the cracks on the sandstone surface are distributed obliquely. 19.89 seconds before peak stress, the cracks begin to propagate, and the inclined cracks on the surface of the sandstone become denser. Near the peak stress, the cracks continue to extend, and bulging becomes more pronounced. During the post-peak residual stage, the oblique cracks on the sandstone surface undergo longitudinal extension and complete penetration, accompanied by intensified skin spalling, a process that takes 33.15 seconds to fully develop.

At a loading rate of 0.20 mm/min, the crack evolution characteristics of sandstone subjected to 0 freeze-thaw cycles are shown in Fig 7. Vertical cracks appear at the upper left position of the sandstone surface at 29.84 seconds before the peak; the cracks begin to expand at 5.32 seconds before the peak; and the cracks extend across the entire sandstone surface at 3.55 seconds before the peak. Complete failure occurs at the peak stress, accompanied by rock bursting, indicating that an increase in loading rate enhances the brittleness of sandstone. The time from the onset of surface cracks to complete cracking is the shortest, which is 38.1% of the time with 0 freeze-thaw cycles at a loading rate of 0.05 mm/min.

**Fig 8:**
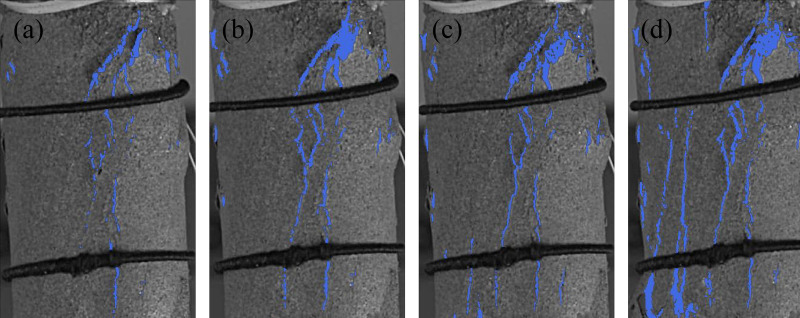
Crack characteristics of sandstone subjected to 70 freeze-thaw cycles at a loading rate of 0.20mm/min. (a) *t*_*p*_-107.3s, (b) *t*_*p*_-15.26s, (c) *t*_*p*_, (d) *t*_*p*_ + 30.23s.

As shown in, under a loading rate of 0.20 mm/min, the crack characteristics of sandstone subjected to 70 freeze-thaw cycles are shown in Fig 8. At 107.3 seconds before the peak stress, under the action of compressive stress, multiple tensile-dominated cracks initiate and propagate along the pre-existing freeze-thaw cracks on the sandstone surface. These cracks begin to extend and expand at 15.26 seconds before the peak, with the cracks developing from top to bottom. Complete failure occurs at 30.23 seconds after the peak. Compared to sandstone with no freeze-thaw cycles at a loading rate of 0.20 mm/min, the sandstone in this case exhibits more severe fragmentation, yet its fragmentation level is still lower than that of sandstone subjected to 70 freeze-thaw cycles at a loading rate of 0.05 mm/min.

## 4. AE characteristic parameters

### 4.1 Crack evolution analysis based on AF and RA

The ratio of AF to RA, denoted as *k*, serves as a criterion for distinguishing tensile and shear cracks in rocks. Typically, tensile cracks correspond to AF/RA > *k*, while shear cracks correspond to AF/RA < *k*. The variations in AF and RA can be used to analyze crack propagation patterns and failure characteristics of rocks under different conditions, providing additional validation for the findings in Section 3.3. The calculation formulas for AF and RA are as follows [42]:


$$\begin{array}{c} AF\;(kHz)=\frac{\text{the\textasciitilde{}hit\textasciitilde{}count}}{\text{duration}} \end{array}$$ (6)

$$\begin{array}{c} RA\;(ms/V)=\frac{\text{the\textasciitilde{}rise\textasciitilde{}time}}{\text{amplitude}} \end{array}$$ (7)

Numerous scholars [43,44] have studied the crack mechanisms of rocks using resonant sensors and found that the value of k is between 80 and 90. Therefore, this paper adopts k = 85 as the threshold to differentiate between different crack types.

**Fig 9:**
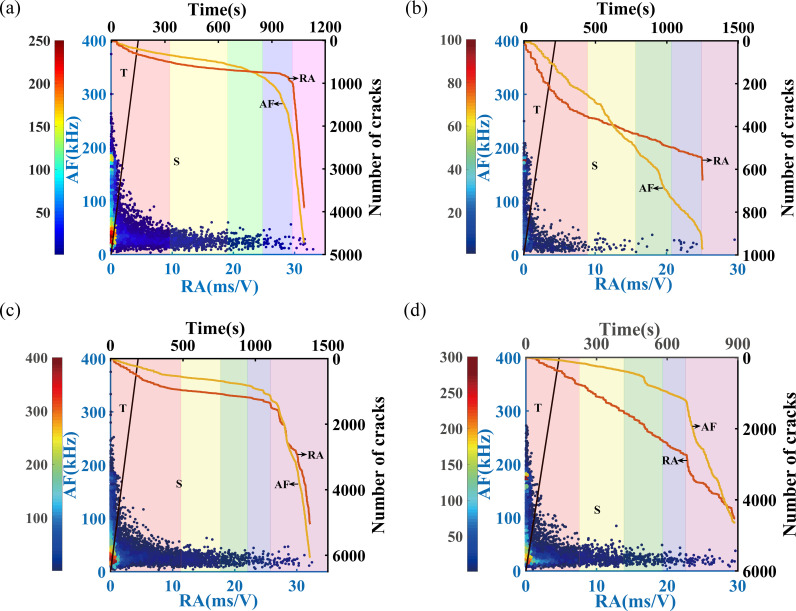
Variation law of RA and AF in sandstone with different freeze-thaw cycles under a loading rate of 0.05mm/min. (a) FT0, (b) FT30, (c) FT50, (d) FT70. ‘T’ refers to the tensile crack area, and ‘S’ refers to the shear crack area. The blue areas signify sparse distribution of scattered points, while the red areas indicate a dense concentration. The two curves shown respectively illustrate the cumulative count of tensile and shear cracks as a function of time.

**Fig 10:**
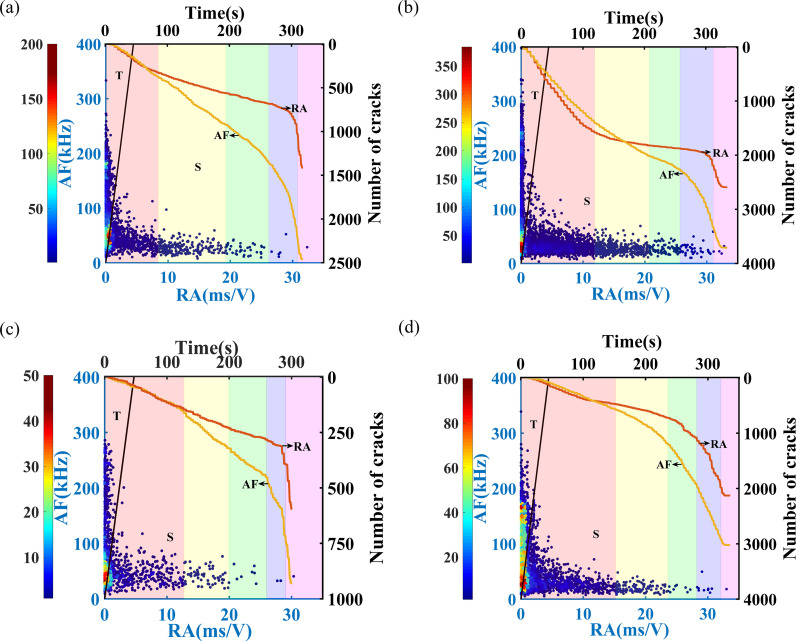
Variation law of RA and AF in sandstone with different freeze-thaw cycles under a loading rate of 0.20mm/min. (a) FT0, (b) FT30, (c) FT50, (d) FT70. The detailed illumination of Fig.10 is the same as that of Fig.9.

The variations of AF and RA in sandstone subjected to different freeze-thaw cycles at loading rates of 0.05 mm/min and 0.20 mm/min are depicted in Fig 9 and Fig 10, respectively. According to the density statistics method, RA and AF are divided into micro-grids, and then the number of scattered points is counted and color-coded according to their density. Distinct background colors are used to represent the different stages of uniaxial compression in sandstone, including the compaction stage, elastic deformation stage, stable crack propagation stage, unstable crack propagation stage, and post-peak residual stage. This approach is also applied in the subsequent figures. As illustrated in Fig 9 and Fig 10, the majority of the densely clustered red regions are located within the tensile crack zones.

With the increase in freeze-thaw cycles, the gap between the cumulative numbers of shear and tensile cracks widens in the early phase of loading, the density of the shear zone increases, and the proportion of shear cracks grows when it is completely destroyed. At a loading rate of 0.05 mm/min, as the number of freeze-thaw cycles increases, the surge in the number of tensile cracks occurs progressively later. In sandstone that has not undergone freeze-thaw cycles, the number of tensile cracks surpasses that of shear cracks during the stable crack propagation stage. However, in sandstone subjected to 50 or 70 freeze-thaw cycles, this crossover occurs only in the post-peak residual stage. Specifically, for sandstone with 50 freeze-thaw cycles, the number of tensile cracks exceeds that of shear cracks 171 seconds before complete failure, whereas for sandstone with 70 freeze-thaw cycles, this occurs 40 seconds prior to complete failure.

As the loading rate increases, the area of high-density colors in the tensile region expands, and the proportion of tensile cracks rises. For sandstone subjected to a loading rate of 0.20 mm/min, the initial number of shear cracks slightly exceeds that of tensile cracks, but tensile cracks begin to dominate during the compaction or elastic deformation stage. During the unstable crack propagation stage, the growth rate of both tensile and shear cracks accelerates. By the time complete failure occurs, the crack development within the sandstone is largely complete, and the difference between the number of tensile and shear cracks decreases as the loading rate increases.

As shown in Fig 9(d) and Fig 10(a), the results of the sandstones subjected to 0 freeze-thaw cycles at a loading rate of 0.2 mm/min and 70 freeze-thaw cycles at a loading rate of 0.05 mm/min exhibit a significant contrast in terms of the proportion and difference in the number of tensile and shear cracks, as well as the time at which the gap between them becomes more pronounced. In the former case, tensile cracks begin to dominate around 70 seconds, and at complete failure, the number of tensile cracks is 1.67 times that of shear cracks. In contrast, in the latter case, shear cracks dominate from the initial compaction phase through the post-peak residual phase, with tensile cracks only surpassing shear cracks approximately 40 seconds before complete failure.

### 4.2 Variation of AE events rate

**Fig 11:**
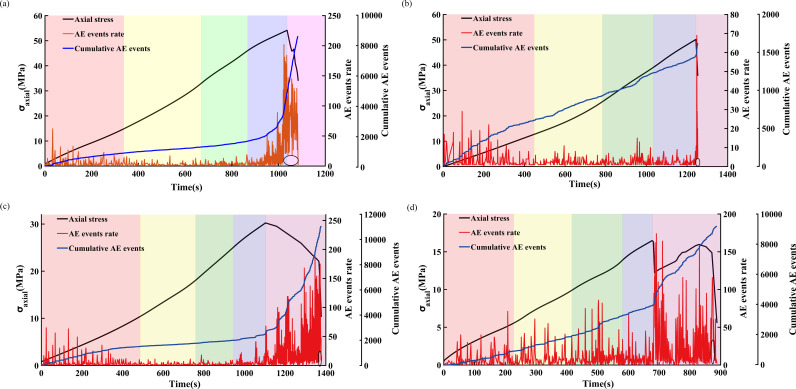
Variation law of AE events rate of sandstone under different freeze-thaw cycles at a loading rate of 0.05 mm/min. (a) FT0, (b) FT30, (c) FT50, (d) FT70. The black circle in the figure indicates the phenomenon of low AE events rate missing.

**Fig 12:**
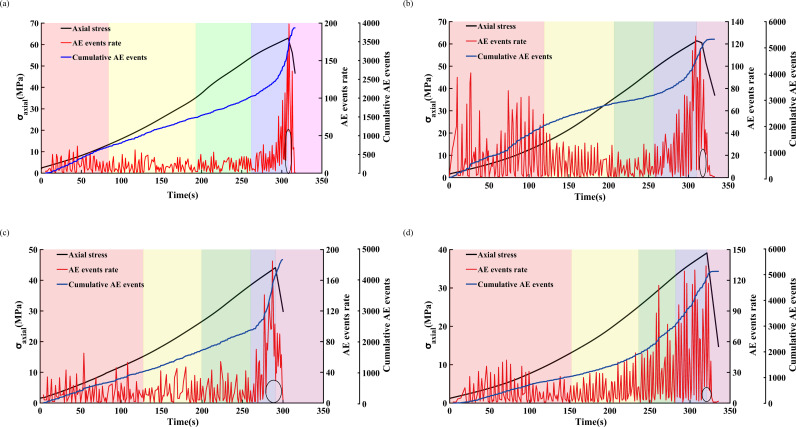
Variation law of AE events rate of sandstone under different freeze-thaw cycles at a loading rate of 0.05 mm/min. (a) FT0, (b) FT30, (c) FT50, (d) FT70. The black circle in the figure indicates the phenomenon of low AE events rate missing.

The AE threshold is set at 10 mV. The AE events rate, calculated as the number of AE signals surpassing the preset threshold per second, signifies the frequency of AE signals. By analyzing the AE events rate, this metric can indicate the internal fracturing of sandstone. Fig 11and Fig 12 illustrate the variation in AE events rate for sandstone subjected to different freeze-thaw cycles at loading rates of 0.05 mm/min and 0.20 mm/min, respectively.

In this study, the level of internal damage and the failure activity within the specimens are assessed by comparing the proportion of each sandstone’s AE events rate to its peak AE events rate.

During the crack compaction stage, the AE events rate is high. In contrast, the AE events rate is low and relatively stable during the elastic deformation stage and the crack stable propagation stage. Prior to the failure instability of the sandstone, the AE events rate begins to increase. And it continues to rise at peak stress and throughout the residual stage after the peak. Except for the sandstone loaded at 0.05 mm/min after 50 freeze-thaw cycles, the peak events rate is all generated at the peak stress. This exception might be due to the heterogeneity of the rocks. In sandstone subjected to various freeze-thaw cycles and loading rates, the phenomenon of low AE events rate loss appears to varying degrees before complete failure, as shown by the black line in the lower right corner of Fig 11and Fig 12. Therefore, the lack of low AE events rate can be used as an index to predict sandstone cracking and instability under different freeze-thaw cycles and loading rates.

Regarding freeze-thaw cycles, the AE events rate of unfrozen sandstone is the lowest during the crack compaction stage. As the number of freeze-thaw cycles increases, more cracks are generated in the sandstone, leading to a gradual rise in the AE events rate. At this stage, the AE event rate with 0 freeze-thaw cycles accounts for about one-fifth of the peak event rate, whereas it accounts for one-third of the peak event rate for 70 freeze-thaw cycles. During the elastic deformation stage and the crack stable propagation stage, the relative displacement of particles at the particle interface produces low event AE rate events, and the duration of these events decreases with the increase in freeze-thaw cycles. Sandstone that demonstrates a significant increase in AE events rate during the accelerated crack growth is more likely to be completely destroyed shortly after reaching the peak stress. With the increase in the number of freeze-thaw cycles, a large number of high AE events rate are produced in the post-peak residual stage, with a significant increase of the cumulative AE events.

The increase in the loading rate reduces the number of AE signals, shortens the time to achieve complete failure, resulting in a generally lower cumulative number of AE events compared to sandstone with a low loading rate. Sandstone subjected to a high loading rate exhibits a higher AE event rate with higher AE activities in each stage and a shorter duration of the stationary period. Under a high loading rate, the AE events rate is high in the elastic stage, and the process of internal crack compaction, development, and expansion is relatively rapid. In the post-peak residual stage, due to its longer duration for sandstones at a low loading rate, internal cracks develop, merge, and coalesce to form macroscopic cracks, and the cumulative number of AE events increases progressively. Under high loading rates, the AE events rate of sandstone increases significantly before reaching the peak stress, peaks at the peak stress, and then the sandstone undergoes an instantaneous brittle failure and instability. Additionally, the phenomenon of missing low AE events rate is more pronounced than under low loading rates.

### 4.3 AE *b*-value variation characteristic

The concept of the AE *b*-value, which stems from seismological research, exhibits the change characteristics that are closely related to the development, expansion, and penetration of rock cracks [45]. The calculation formula of AE is:


$$lgN=a-b\;\;\frac{A_{dB}}{20}$$ (8)

where *N* is the sum of AE data in each time window, A_dB_ is the AE amplitude, and a is the empirical constant.

According to the AE sampling frequency, a sliding window of 50 data points is employed for sampling from a total of 200 samples. The AE *b*-value is then calculated using the least squares method. An increase in the *b*-value indicates that small-scale cracks are predominant within the rock, whereas a decrease in the AE *b*-value suggests the occurrence of large-scale cracks within the rock. When the AE *b*-value fluctuates within a small range, it signifies a steady expansion of cracks within the rock. Conversely, when the AE *b*-value exhibits abrupt fluctuations, it indicates a rapid propagation of cracks within the rock [46].

**Fig 13:**
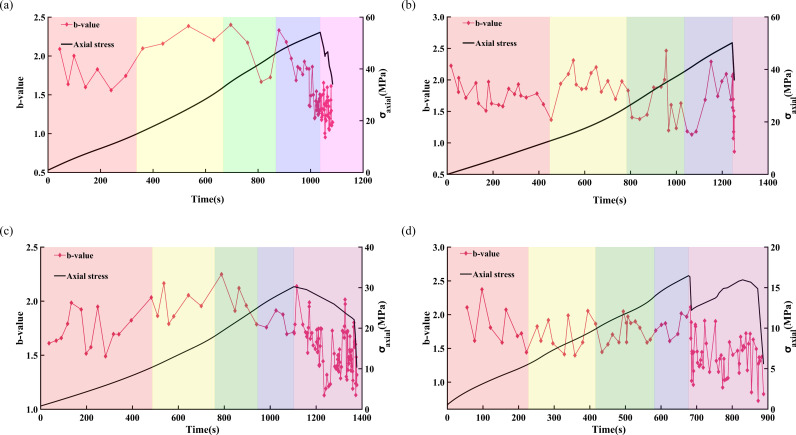
Variation law of AE *b*-value of sandstone under different freeze-thaw cycles at a loading rate of 0.05 mm/min. (a) FT0, (b) FT30, (c) FT50, (d) FT70.

**Fig 14:**
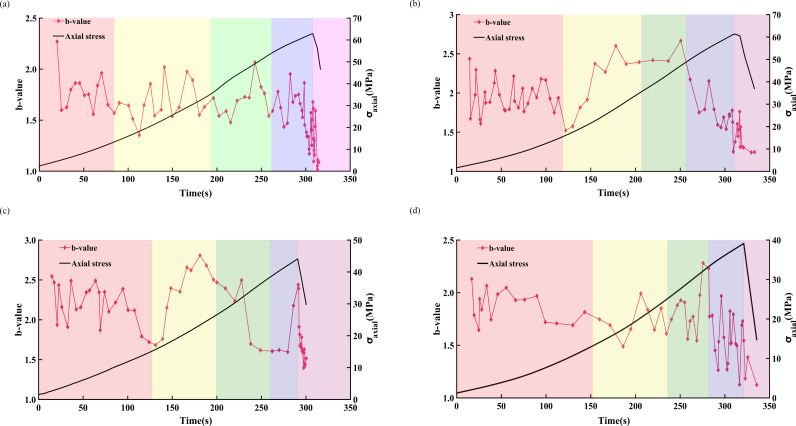
Variation law of AE *b*-value of sandstone under different freeze-thaw cycles at a loading rate of 0.20mm/min. (a) FT0, (b) FT30, (c) FT50, (d) FT70.

Fig 13 and Fig 14 illustrate the variations in the AE *b*-value and stress-strain relationship of freeze-thawed sandstone under loading rates of 0.05 and 0.20 mm/min, respectively. The overall trend of a gradually decrease in AE *b*-value signifies the process of crack initiation, development, and coalescence within the rock.

During the compaction phase, the AE *b*-value exhibits frequent fluctuations and an overall downward trend, indicating that pre-existing cracks within the sandstone are gradually being compacted while new cracks form. Upon entering the elastic deformation stage, the fluctuations in the AE *b*-value stabilize and generally trend upwards, suggesting the formation and expansion of new cracks within the sandstone. For sandstone subjected to low freeze-thaw cycles and low loading rates, a significant decrease in the AE *b*-value is first observed during the stable crack propagation stage. In contrast, other sandstones only exhibit a marked reduction in the AE *b*-value during the unstable extension of cracks, marking the onset of crack propagation within the sandstone. In the unstable extension phase of cracks, both sandstones subjected to low freeze-thaw cycles and those with high loading rates consume a large amount of strain energy, leading to the expansion and coalescence of internal cracks within the sandstone, and a sharp decrease in the AE *b*-value. Upon reaching the peak stress, the AE *b*-value continues to decrease. In the post-peak residual stage, the internal cracks in sandstone extend and interconnect. Sandstone with a high loading rate is completely destroyed immediately after the peak value, and low *b*-values appear frequently, with a significant decrease in the AE *b*-value. In contrast, under the condition of a loading rate of 0.05 mm/min, the duration of the high-frequency decline in the AE *b*-value signal is obviously increased, for example, in sandstone subjected to 50 and 70 freeze-thaw cycles. At the point of complete failure, cracks rapidly coalesce, and the AE *b*-value reaches its lowest point.

## 5. Discussion

### 5.1 Exponential decay model under dual-factor coupling conditions

**Fig 15:**
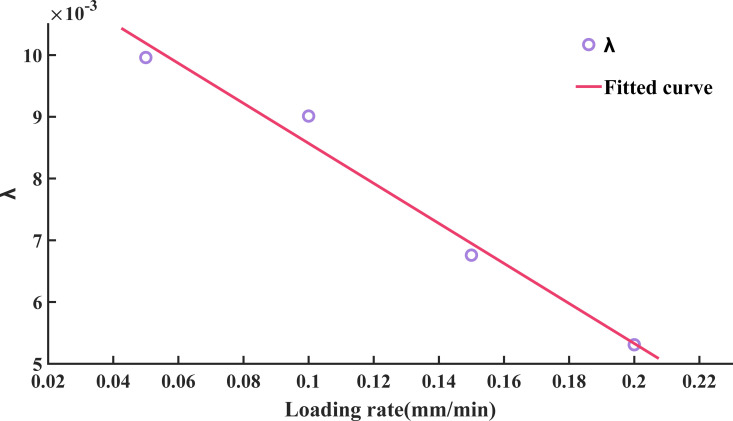
Variation law of attenuation constant of sandstone with different loading rates.


Huang et al. [2,41] proposed the exponential decay model for rocks subjected to freeze-thaw cycles, while Meng et al. [47] established the corresponding exponential decay model for sandstone under different dynamic fracturing rates. Building on the experimental findings presented in Section 3.2, this Section seeks to further assess the applicability of the exponential decay model to freeze-thawed sandstones subjected to varying loading rates [48]. Fig 15 illustrates the variation in the sandstone attenuation constant under different loading rates and further conducts a linear fitting, as shown by:

$$\lambda =-0.03242\times v+0.01181,R^{2}=0.9681$$ (9)

Where v is the loading rate, mm/min.

Substituting Eq. (9) into Eq. (1), we can get:


$$I_{\text{n}}=I_{0}e^{(0.03242\nu -0.01181)n}$$ (10)

**Fig 16:**
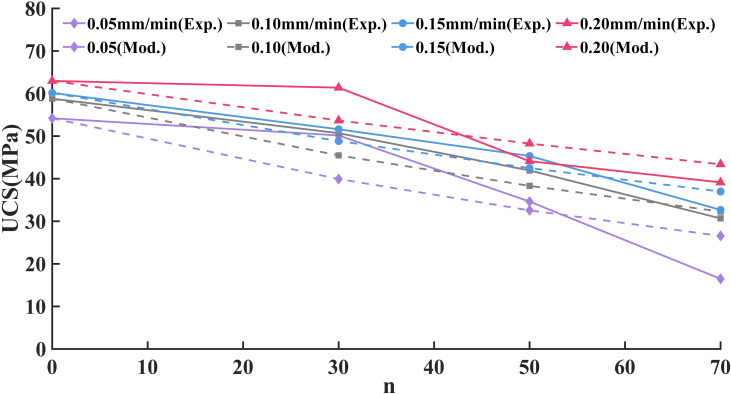
Comparison of UCS tests and models of sandstone under different loading rates and freeze-thaw cycles.

The experimental results were substituted into the exponential decay model (10), and the model showed a good fit, as shown in Fig 16. Thus, Eq. (10) can be utilized to estimate the variations in UCS after differing numbers of freeze-thaw cycles at a loading rate between 0.05 and 0.20 mm/min. This not only compensates for the limitation of testing specific freeze-thaw cycles but also enhances the reliability of predictions regarding the UCS of rocks under freeze-thaw conditions.

To assess the durability of rocks post freeze-thaw cycles, Mutlutürk et al. [40] introduced the concept of half-life, $N_{1/2}$, for rocks. This term refers to the number of freeze-thaw cycles a rock undergoes until its strength is reduced by half. The half-life is an important metric for evaluating a rock’s resistance to frost, as depicted in:


$$I_{1/2}=\frac{1}{2}I_{0}$$ (11)

By combining Eqs. (1) and (11), the half-life $N_{1/2}$ of the freeze-thawed sandstone discussed in this paper is determined as follows:


$$N_{1/2}=\frac{\ln2}{\lambda }$$ (12)

Similarly, if Eq. (9) is substituted into Eq. (12), we can get:


$$N_{\text{1/2}}=\frac{\ln2}{\left(-0.03242\nu +0.01181\right)}$$ (13)

Using the aforementioned formula, we can ascertain the requisite number of freeze-thaw cycles for the strength of sandstone to degrade to half its initial value within the loading rate spectrum of [0.05, 0.20] mm/min. Our calculations reveal that at loading rates of 0.05, 0.10, 0.15, and 0.20 mm/min, the sandstone’s strength reduces to half after 70, 77, 105, and 131 freeze-thaw cycles, respectively. Therefore, by considering the number of equivalent freeze-thaw cycles per year in the actual project’s climate, we can approximate the time it would take for the sandstone to reach its half-life.

### 5.2 Damage mechanism under dual-factor coupling

The results presented in Sections 3.1 and 3.3 demonstrate that both freeze-thaw cycles and loading rates significantly affect the mechanical properties and crack characteristics of fully water-saturated sandstone. With an increasing number of freeze-thaw cycles, the peak strength of sandstone decreases owing to the freezing and expansion forces, which weaken the cementation of internal particles. As shown in Section 4.2, a higher number of freeze-thaw cycles prolongs the post-peak residual phase, resulting in a greater number of high AE events rate, while the growth rate of cumulative AE events slows after reaching an inflection point. Section 4.3 further reveals that the duration of successive decreases in the AE *b*-value also extends. Collectively, these findings suggest that an increase in freeze-thaw cycles enhances the ductility of sandstone. Furthermore, as temperatures drop during freeze-thaw cycles, the temperature gradient between the surface and interior of the sandstone induces differential shrinkage, causing tensile stress on the sandstone surface. As demonstrated in Section 3.3, the combined action of tensile stress and freezing expansion forces leads to particle detachment on the sandstone surface after multiple freeze-thaw cycles. Based on the findings in Sections 3.3 and 4.1, it can be concluded that under lower loading rates, the failure mode of sandstone subjected to different freeze-thaw cycles is characterized by tensile-shear mixed damage. However, as the loading rate increases, the failure mode gradually transitions to a mixed damage mode dominated by tensile failure.

An increase in loading rate reduces the efficiency with which sandstone converts absorbed energy into dissipated energy, resulting in delayed onset of damage, a significant increase in peak strength, and a shortened duration of the post-peak residual phase. Sandstone that has not undergone freeze-thaw cycles exhibits more extensive cracking and greater damage at higher loading rates, leading to enhanced brittleness. As demonstrated in Section 3.1, the influence of loading rate on the UCS of freeze-thawed sandstone, and as revealed in Section 3.2, the variation in the attenuation constant λ, indicate that within the loading rate range of [0.05, 0.20] mm/min, increased loading rate can mitigate the damaging effects of freeze-thaw cycles on sandstone to some extent. In terms of RA, AF, AE events rate, and AE *b*-value evolution, higher loading rates diminish the impact of freeze-thaw cycles on AE signals. However, fracture characteristics indicate that the rupture evolution of sandstone is notably governed by freeze-thaw damage. This phenomenon occurs because the freezing and expansion forces during multiple freeze-thaw cycles cause microcracks to proliferate, merge, and propagate within the water-saturated sandstone, leading to the formation of multiple macroscopic fissures across varying loading rates. Consequently, the influence of water content on sandstone stability during freeze-thaw cycles is of critical importance.

AE systems are widely utilized as non-destructive monitoring tools for predicting instability damage in sandstone. Previous research has predominantly concentrated on predictive indicators under single-variable conditions, with limited studies addressing multivariable scenarios. As revealed in Sections 4.2 and 4.3, both the AE events rate and the AE *b*-value remain effective predictors for freeze-thawed sandstones subjected to various loading rates. However, the AE events rate proves to be a more reliable predictor compared to the AE *b*-value, especially at high loading rates, where the absence of low AE events rates is more pronounced for sandstone.

## 6. Conclusions

In this study, uniaxial compression tests combined with AE monitoring were conducted on water-saturated sandstone subjected to varying loading rates and freeze-thaw cycles. By examining the mechanical properties, crack characteristics, and AE signals, the damage evolution and crack mechanisms of sandstone under the combined influence of loading rate and freeze-thaw processes were investigated. The primary conclusions drawn from this analysis are as follows:

- An increase in freeze-thaw cycles combined with a decrease in loading rate leads to a reduction in the UCS and *E* of sandstone, with strain initially decreasing and then increasing. This process also enhances the ductility of the sandstone. The attenuation constant λ decreases as the loading rate increases, indicating that higher loading rates mitigate the strength deterioration caused by freeze-thaw cycles to some extent. The relationship between UCS and the combined effects of loading rate and freeze-thaw cycles can be described by an exponential decay model.
- With the increase of freeze-thaw cycles and the decrease of loading rate, the time from the initiation of cracks to the complete failure of the sandstone surface increases. Upon complete failure, the number of tensile cracks in sandstone exceeds that of shear cracks. The proportion of shear cracks initially increases with the increase of freeze-thaw cycles but then decreases with the increase of loading rate. As the number of freeze-thaw cycles increases, the failure mode of sandstone transitions to a blend of tensile and shear failure. However, with the increase of loading rate, the sandstone predominantly exhibits tensile failure.
- In the stages of compaction, crack propagation, and post-peak residual deformation, the rate of AE events increases progressively, and the fluctuation range of the *b*-value becomes wider. As the loading rate escalates, the AE events rate throughout the entire loading cycle becomes more active, with the occurrence of high AE events rates advancing to just before the peak load. This is attributed to the rapid compaction process within the sandstone, leading to a reduction in the initial *b*-value. Low AE events rate omissions and significant *b*-value declines serve as precursors for identifying instability damage in freeze-thawed sandstones across varying loading rates. Among these indicators, the absence of low AE events rates proves to be a more effective predictive method.

Although this study reveals the effects of freeze-thaw cycles and loading rates on the mechanical properties of sandstone, certain limitations remain. For instance, while the exponential decay model effectively captures the attenuation of UCS, its applicability requires further validation. In addition, the mechanisms underlying the influence of different water contents, freeze-thaw cycle temperatures, and cycle numbers on rock damage evolution under multi-physical field coupling still require systematic research. Future research should focus on these critical factors to achieve a more comprehensive understanding of how freeze-thaw cycles influence the long-term stability of rock masses.
